# Loss of bronchoprotection to Salbutamol during sputum induction with hypertonic saline: implications for asthma therapy

**DOI:** 10.1186/s13223-018-0256-7

**Published:** 2018-05-10

**Authors:** Hongyu Wang, Melanie Kjarsgaard, Terence Ho, John D. Brannan, Parameswaran Nair

**Affiliations:** 1 Firestone Institute for Respiratory Health, St. Joseph’s Healthcare, 50 Charlton Avenue East, Hamilton, ON L8N 4A6 Canada; 2 Department of Medicine, McMaster University, Hamilton, ON Canada; 3 John Hunter Hospital, Newcastle, NSW Australia

**Keywords:** Hypertonic saline, Sputum induction, Bronchoconstriction, Long-acting beta-agonists, Asthma, COPD

## Abstract

**Background:**

Sputum induction with hypertonic saline in obstructive airway diseases is generally safe. However, saline induces bronchoconstriction in some patients despite pre-medication with Salbutamol. Our objectives were to investigate the predictors of failure of Salbutamol to protect against saline-induced-bronchoconstriction in patients with asthma and COPD and to evaluate implications for asthma therapy.

**Methods:**

Retrospective survey on a database of 3565 patients with obstructive airway diseases who had sputum induced with hypertonic saline. The effect of baseline FEV_1_, bronchitis and concomitant medication on saline-induced-bronchoconstriction (≥ 15% drop in FEV_1_) were examined by logistic regression analysis. A subgroup had this re-examined 8–12 weeks after decreasing long-acting-beta-2-agonist dose or after adding Montelukast, which included an assessment of mast cell activity in sputum.

**Results:**

222 (6.2%) patients had saline-induced-bronchoconstriction despite pre-treatment with inhaled Salbutamol. Baseline airflow obstruction (FEV_1_% predicted < 60% OR 3.29, p < 0.001) and long-acting-beta-agonist use (OR 2.02, p = 0.001), but not bronchitis, were predictors of saline-induced-bronchoconstriction, which decreased when long-acting-beta-agonist dose was decreased. Refractoriness to subsequent bronchodilation was associated with mast cell activity and was attenuated by Montelukast.

**Conclusion:**

Sputum induction with saline provides information on bronchitis and additional physiological data on tolerance to beta-agonists and mast cell activity that may have implications for clinical therapy.

## Background

Hypertonic saline nebulization is a relatively non-invasive procedure to collect sputum for airway diseases even in the presence of moderate to severe airflow obstruction [1]. Occasionally, despite pre-medication with Salbutamol, saline-induced bronchoconstriction (SIB) occurs. This may be related to baseline airflow obstruction, increased airway hyperresponsiveness (AHR), or lowered sensitivity to β_2_-agonists [2–5]. The loss of bronchoprotection is considered to be primarily due to β_2_-receptor downregulation and desensitization [3], and the refractoriness to subsequent bronchodilation with Salbutamol (i.e. recovery time) is considered to be mediated partly by leukotrienes and thus reflecting mast cell activity [6].

The objectives of this retrospective cross-sectional survey were to determine predictors of SIB in a large cohort of patients with airway disease and to illustrate the wealth of information on airway physiology that could be obtained during the process of sputum induction. As proof of principle, we also evaluated the effect of LABA-dose reduction and leukotriene antagonism on SIB in a non-randomised observational study.

## Methods

Data were collected from a computerized database of induced sputum cell counts from January, 2004 to January, 2008 at the Firestone Institute for Respiratory Health in Hamilton, Ontario. The database contained the following information: age, gender, post-bronchodilator spirometry, FEV_1_ after each concentration increment of saline (3, 4, 5%, each for 7 min), and after subsequent administration of Salbutamol, sputum cell counts, referring physician diagnosis, indication for the test, and current relevant medications. Three groups of patients were included in the analysis: current asthma with or without associated chronic airflow limitation, possible asthma (when the referring physician was not certain of the diagnosis), and non-asthmatic COPD. A diagnosis of asthma was based on previous evidence of reversible airflow limitation (an increase in FEV_1_ ≥ 15% and ≥ 200 ml from the pre-bronchodilator value) or airway hyper responsiveness (a provocative concentration of methacholine causing a > 20% fall in FEV_1_ < 8 mg/ml). COPD was indicated by a post-bronchodilator FEV_1_/VC < 70%, and history of cigarette smoking or smoker’s inclusions within macrophages.

FEV_1_ and FEV_1_/VC were measured according to ATS standards 10 min after subjects received 200 μg of Salbutamol. Sputum was induced and processed according to previously published methods [7]. Saline-induced bronchoconstriction was defined as a ≥ 15% drop in FEV_1_ from pre-saline values at any of the concentrations of saline. Prior to induction, subjects did not withhold their regular medications, including long-acting bronchodilators, as per our protocol. Metachromatic cells were stained using toluidine blue in a subset of patients who also had their tryptase measured in cell-free sputum supernatant by ELISA. Methacholine provocation test results (by the tidal breathing method; [8]) were available for 56 subjects, where bronchodilating medications were withheld as per guidelines [9]. Two subsets of patients with SIB were re-evaluated 8–12 weeks after either reducing their dose of LABA by half (n = 36) or after treating them with Montelukast 10 mg daily (n = 20), as part of their routine clinical management. The study was approved by the Research Ethics Board of St. Joseph’s Healthcare, Hamilton. Descriptive statistics were used to summarize the baseline characteristics of the patients. Multivariate logistic regression was used in forward and backward stepwise approach to determine predictors of SIB (PASW Statistics 18, SPSS, Chicago, IL).

## Results

3565 patients had sputum induced for the assessment of bronchitis (Table 1), of whom 222 (6.2%) had a ≥ 15% fall in FEV_1_. Overall, the predictors of Salbutamol failing to protect against SIB were the use of LABA (OR 2.02, 95% CI 1.32–3.01, p = 0.001), high doses of ICS (OR 1.85, 95% CI 1.11–3.09, p = 0.02), and baseline airflow obstruction (FEV_1_/VC < 70%; OR 2.08, 95% CI 1.40–3.10, p < 0.001) and FEV_1_ predicted < 60% (OR 3.29, 95% CI 2.06–5.26, p < 0.001). The presence or type of bronchitis were not predictors (Table 2). In the subset of patients who had a concurrent methacholine test (n = 56), a PC_20_ methacholine of < 2 mg/ml was significantly associated with SIB (OR 7.50, 95% CI 2.04–22.66, with p = 0.002 by Fisher’s exact test).

**Table 1:** Baseline characteristics of patients

	Patients, no. (%)			
	All patients n = 3565	Asthma n = 2013	Possible asthma n = 157	Non-asthmatic COPD n = 1395
FEV_1_ ↓ > 15% (%)	222 (6.2)	152 (7.5)	22 (14.0)	48 (3.4)
Male sex (n, %)	1569 (44.0)	708 (40.3)	100 (63.7)	761 (54.5)
Age year (mean, SD)	54 (17)	47 (17)	44 (13)	66 (11)
ICS (n, %)	1957 (54.9)	1661 (82.5)	102 (65)	194 (13.9)
LABA (n, %)	2426 (68)	1381 (68.6)	51 (32.5)	994 (71.3)
OCS (n, %)	174 (4.9)	135 (6.7)	8 (5.1)	31 (2.2)
NB (n, %)	328 (16.6)	106 (5.3)	15 (9.6)	207 (14.8)
EB (n, %)	592 (13.8)	534 (26.5)	20 (12.7)	37 (2.7)
FEV_1_ % (mean, SD)	62.5 (45.5)	68.7 (33.6)	78.4 (22.5)	59.8 (40.8)
FEV_1_/VC % (mean, SD)	64.4 (37.0)	68.7 (43.7)	72.2 (24.6)	54.6 (34.5)

*ICS* inhaled corticosteroid, *NB* neutrophilic bronchitis, *EB* eosinophilic bronchitis, *OCS* oral corticosteroid, regular or intermittent, *LABA* long-acting β-agonist

Eosinophilic bronchitis (EB) was defined as percentage of sputum eosinophils ≥ 3%. Neutrophilic bronchitis (NB) was defined as a total cell count ≥ 15 million cells/g of sputum and proportion of neutrophils ≥ 64%

**Table 2:** Predictors of saline-induced bronchoconstriction

	All patients, n = 3565			
	No.	FEV_1_ fall > 15%, no. (%)	OR (95% CI)	p value
High ICS dose	785	84 (10.7)	1.85 (1.11–3.09)	0.019
LABA use	2426	142 (10.0)	2.02 (1.32–3.10)	0.001
FEV_1_ < 60% predicted	596	93 (15.6)	3.29 (2.06–5.26)	< 0.001
FEV_1_/VC < 70%	1165	149 (12.8)	2.08 (1.40–3.10)	< 0.001

*ICS* inhaled corticosteroid, *LABA* long-acting β-agonist

Of the 36 asthmatics who had their dose of LABA halved, 25 (69%) did not demonstrate SIB during a second sputum induction done 8–12 weeks after the dose adjustment. Sputum mast cell activity was measured in 20 subjects who demonstrated refractoriness to bronchodilation after saline induction (mean time for FEV_1_ to return to within 5% of pre-induction baseline was 38 ± 6 min), and this revealed that metachromatic cells (2.2 ± 0.8% vs. 0%) and tryptase (5.6 ± 1.8 vs. 0.8 ± 1.4 pg/ml) were both increased when compared to reference values [7]. In 14 (70%) of these patients, the addition of Montelukast for 8–12 weeks resulted in reduced SIB and a faster recovery of FEV_1_ (mean time 17 ± 8 min).

## Discussion

We confirmed previous observations that baseline airflow limitation and airway hyperresponsiveness to a direct stimulus such as methacholine can predict the loss of bronchoprotection to Salbutamol during saline induction [2], but also established that LABA use is a risk factor in a mixed population of obstructive airway diseases. LABA appears to cause these effects by way of receptor tolerance, [10–13]. β-receptor tolerance of airway smooth muscle cells can manifest as reduced bronchodilation, whereas for mast cells may manifest with an increased propensity to release inflammatory mediators [14]. For those on high-dose LABA, we found that reducing the dose by half led to the resolution of SIB in almost 70% of subjects. This suggests that it is important to recognize this phenomenon and to reduce the dose of LABA rather than increasing it in those patients with asthma who may have tolerance either to its bronchodilator or bronchoprotective effects.

Although we did not observe the cellular nature of bronchitis in our study to be a predictor of tolerance to SIB, there is evidence to suggest that the tolerance to bronchoprotection occurs more readily to indirect rather than to direct bronchconstrictive agents suggesting that airway inflammation may contribute to this phenomenon. One possible explanation that may account for these previous findings is airway mast cell activity that we do not routinely assess in quantitative sputum cell counts. This is supported by a study demonstrating that regular short-acting β-agonist leads to higher sputum levels of tryptase and metachromatic cells (mostly basophils), and an enhanced early and late asthmatic response [14]. Our findings corroborate a role for mast cells, as we showed less SIB and a more rapid recovery of FEV_1_ after SIB with the use of Montelukast in those with elevated sputum tryptase and metachromatic cells.

The major limitation of this study is the retrospective design of this study, which prevents the establishment of a causal relationship. LABA dose was not available for all patients and this study was not powered to detect differences between Formoterol and Salmeterol. Non-respiratory medications which may impact relevant pathways, including β-adrenergic blockers (e.g. eye drops, tablets) were not recorded within this retrospective survey. Finally, the interventions were not evaluated in a placebo, controlled, randomised trial design thus limiting interpretation of the efficacy that we observed.

## Conclusions

In summary, we report two clinically relevant findings regarding airway pathophysiology that could be gleaned during the process of sputum induction using hypertonic saline: first, failure of Salbutamol to protect against saline-induced bronchoconstriction should raise suspicion of tolerance to the bronchoprotective effect of β-agonists. Such patients may benefit from reducing the dose or frequency of use of LABA. Second, a prolonged recovery time (refractoriness) of FEV_1_ following saline bronchoconstriction may indicate mast cell activity and may suggest that these are patients who may respond to mast-cell directed therapy or therapy directed against products of mast cells such as leukotriene receptor antagonists. It would be relevant to examine this phenomenon in relation to the mast cell signatures that have recently been reported using transcriptomic analysis of sputum [15, 16]. It is important to test both LABA dose reduction to improve β-agonist sensitivity and mast-cell targeted therapy to improve refractoriness to hyperosmolar stimuli induced bronchoconstriction in placebo-controlled randomised clinical trials.

## Abbreviations

SIB: saline-induced bronchoconstriction
AHR: airway hyperresponsiveness
LABA: long-acting β-agonist
ICS: inhaled corticosteroid
EB: eosinophilic bronchitis
NB: neutrophilic bronchitis
